# Do the human gut metagenomic species possess the minimal set of core functionalities necessary for life?

**DOI:** 10.1186/s12864-020-07087-8

**Published:** 2020-09-30

**Authors:** Matteo Soverini, Simone Rampelli, Silvia Turroni, Patrizia Brigidi, Elena Biagi, Marco Candela

**Affiliations:** Department of Pharmacy and Biotechnology, Unit of Microbial Ecology of Health, University of Bologna, Via Belmeloro 6, 40126 Bologna, Italy

**Keywords:** Gut microbiome, Metagenomic assembled genomes, Uncultured metagenomic species, Minimal bacterial genome

## Abstract

**Background:**

Advances in bioinformatics recently allowed for the recovery of ‘metagenomes assembled genomes’ from human microbiome studies carried on with shotgun sequencing techniques. Such approach is used as a mean to discover new unclassified metagenomic species, putative biological entities having distinct metabolic traits.

**Results:**

In the present analysis we compare 400 genomes from isolates available on NCBI database and 10,000 human gut metagenomic species, screening all of them for the presence of a minimal set of core functionalities necessary, but not sufficient, for life. As a result, the metagenome-assembled genomes resulted systematically depleted in genes encoding for essential functions apparently needed to support autonomous bacterial life.

**Conclusions:**

The relevant degree of lacking core functionalities that we observed in metagenome-assembled genomes raises some concerns about the effective completeness of metagenome-assembled genomes, suggesting caution in extrapolating biological information about their metabolic propensity and ecology in a complex environment like the human gastrointestinal tract.

## Background

Integral to the human biology, the Gut Microbiome (GM) is a key determinant of our health and its dysbiotic variations have been associated with several inflammatory diseases [1]. Species-level variation in GM has been indicated as an emergent factor to be considered both for a better understanding of the biology of the GM-host mutualism [2] and for a refined evaluation of the individual health risk [3]. However, even if it is perceived as strategic in GM study, the capability of shotgun metagenomics to infer species-level taxonomic and functional information is traditionally limited by the relative paucity of reference genomes. Indeed, despite the important progresses in culturomics, the degree of unclassified GM diversity at the species level is still very high. An important step forward in this direction has been recently provided by genome-resolved metagenomics, which involves the simultaneous recovery of draft and complete genomes directly from sequenced metagenomes [4]. In particular, this approach consists in a de novo assembly of shotgun metagenomic reads into contigs, which are binned on the basis of coverage and tetranucleotide frequency [5, 6]. This strategy allows the recovery of thousands of new genomes, i.e. the so called ‘Metagenome-Assembled Genomes’ (MAGs), directly from metagenomic reads, considerably expanding the tree of life beyond the limits of cultivability [7]. Recently, Almeida et al. [8] provided a first extensive discovery campaign of MAGs from 13,133 human metagenomic samples. In particular, the Authors successfully characterized 1175 MAGs, the so-called MetaGenomic Species (MGS – entities having a confirmed taxonomic assignation), estimating a median completeness of 96.5 and 0.8% of contamination. Further, additional 893 medium quality MGS were also detected, with median completeness of 77.8 and 1.1% of contamination, increasing the total number of newly uncharacterized bacterial species to 2068. These of these MAGs did not match any bacterial isolate genome included in the Human-specific Reference (HR) [9] and RefSeq databases and thus indicated as new Unclassified MetaGenomic Species (UMGS). 74% of UMGS correspond to entirely novel genomes. 26% of the UMGS belonged to potential new families and 40% to new genera, thus expanding our current knowledge of human bacterial lineage by 281%. The Authors also performed an in-depth functional characterization of 2505 human gut species, 2068 UMGS and 553 isolates from the Human Gut Reference (HGR) database, i.e. gut-specific species from the HR database [8]. Interestingly, UMGS resulted depleted in genes involved in antioxidant activities and redox functions, being conversely enriched in iron-sulfur and ion binding genes. Thus, the Authors concluded that the recovered UMGS corresponded to strict anaerobes, with a distinctive metabolic propensity, well adapted to specific niches of the gastrointestinal tract with particularly low oxygen tension and high iron concentration.

Several research projects have been carried out with the specific purpose to define a ‘minimal genome’ as a model for understanding the basic functions of life [10]. This resulted in the identification of a set of core functionalities necessary for a bacterium to survive and reproduce, as a universal minimal gene set represented in all living systems [11]. In order to explore the efficacy of genome-resolved metagenomics in providing comprehensive biological information on the uncultured members of the human microbiome, here we wondered if UMGS, which now remain bioinformatic entities, possesses the minimal set of core genes necessary – even if not sufficient – for life. To this aim, two publicly available minimal genomes were used as reference to generate a Core gene set of Minimal Functions (CMF), apparently necessary – but not sufficient – for life. Then we attempted to screen both UMGS and isolated NCBI genomes for the presence of genes included in CMF, showing that a remarkable number of UMGS were depleted in essential functionalities generally necessary for autonomous life.

## Results

The aim of the present study was to provide a first screening of UMGS and isolates genomes for a minimal subset of genetic functions (CMF) necessary – but not sufficient - to sustain bacterial life. In order to generate the CMF, two publicly available minimal genomes were downloaded from NCBI website: JCVI-syn 3.0 genome generated by Hutchison et al. [11] and *C. Eth-2.0* genome generated by Venetz et al. [12]. The two genomes were annotated and only the genes assigned with certainty (not being prefixed by *putative* or *hypothetical*) and present in both genomes were retained and used as a reference set for the CMF. The CMF covered the JCVI-syn 3.0 and *C. Eth-2.0* genomes at 91 and 84%, respectively. The CMF mostly includes genes involved in genetic information processing and cytosolic metabolism (Additional file 1). In particular, of the 183 genes included in CMF (Additional file 2), 143 were assigned by KEGG orthology to the genetic information processing pathways, with the functions involved in translation highly represented, including 115 genes among which 44 encode for ribosomal subunits, 20 for aminoacids-tRNA ligases, and 24 for tRNA. Replication and repair are other groups of functions highly represented in the CMF list, including 16 genes encoding for DNA polymerases, gyrases, and topoisomerases among others. Conversely, 35 out of 183 genes are devoted to metabolic functions, including especially carbohydrate metabolic pathways (e.g. glycolysis and gluconeogenesis, galactose metabolism, starch and sucrose metabolism, etc), energy metabolism (including all subunits of ATP synthase), and metabolism of nucleotides. Only two genes included in CMF are exclusively devoted to environmental information processes, and other two to cellular processes. However, 7 out of 183 genes showed multiple functionalities according to their KEGG orthology; for instance, Enolase is involved in metabolism, genetic information processes and environmental information processes, Phosphoglycerate kinase is involved in both metabolism and environmental information process, and two Protein translocase subunits (SecA and SecY) are involved in genetic information processes, environmental information processes and cellular processes.

**Fig. 1:**
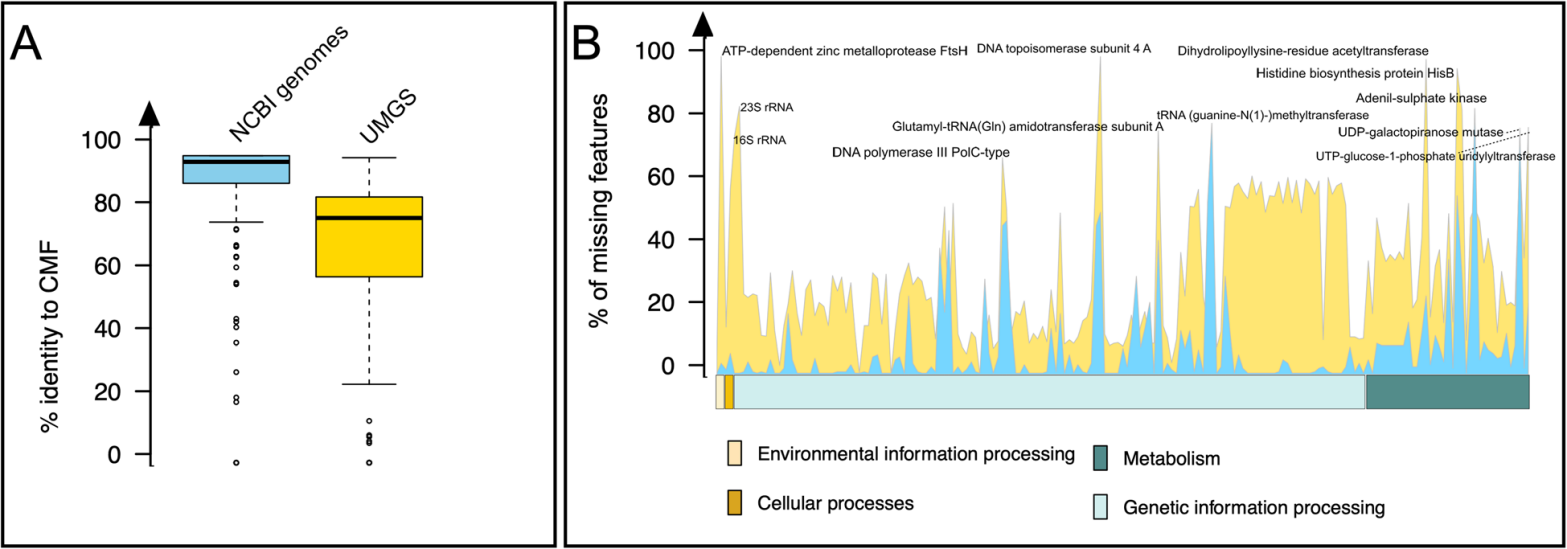
**a** Percentage of genes in NCBI (skyblue) and UMGS (gold) genomes that were included in CMF. NCBI genomes show a significantly greater adherence to CMF (*P* < 0.001, Wilcoxon test). **b** Superimposed distribution of missing CMF genes in NCBI (skyblue) and UMGS (gold) genomes. For each gene included in CMF, the percentage of genomes lacking the correspondent function is plotted. Genes in CMF are clustered according to the functional classes, as in Additional file 1

We scanned both UMGS and isolated NCBI genomes for the presence of genes included in CMF. To this aim, 10,000 human gut metagenome-assembled UMGS were randomly downloaded from the UMGS database generated by Almeida et al. [8], including the 1175 high quality and 893 mid quality UMGS. On the other hand, the 400 NCBI genomes were carefully selected to include a panel of isolates from the human gut which approximate the overall phylogenetic diversity of the ecosystem. Phylogenetic information about the genomes included in this study, and the species included in the selected genomes, are reported in the Additional file 3. Each genome set was then annotated, and for both the NCBI and UMGS genomes, the presence or the absence of each gene included in CMF was verified, generating a binary matrix of CMF presence/absence profiles. For each tested genome, the percentages of adherence to the CMF and the absolute amounts of missing entries were also computed. Our analysis revealed that the NCBI and the UMGS genomes are characterized by a significant different presence of the CMF (*P* < 0.001, Kruskall-Wallis test), with the NCBI genomes showing a higher average representativeness value and a lower standard deviation when compared to UMGS (93.2% ± 2.9 and 67.9% ± 9.5 Standard Deviation for NCBI and UMGS genomes, respectively) (Fig. 1a). Particularly, when comparing the percentage of adherence to CMF between UMGS with a CheckM score equal or greater than 90% (high quality), UMGS with a CheckM score below 90% and NCBI genomes, the latter resulted significantly higher in CMF representation (*P* < 0.001, Kruskall-Wallis test) and, as expected, high quality UMGS showed an higher percentage of adherence to CMF with respect to low quality UMGS (*P* < 0.001, Kruskall-Wallis test) (Additional file 4). In Fig. 1b the overall profile of the missing CMF in NCBI and UMGS genomes is reported. No differences in CMF hits in the UMGS genomes assigned at the different phylogenetic levels were obtained (data not shown). The CMF were found generally less represented in UMGS, with a total of 45 genes lacking in more than 50% analyzed genomes, with respect to the NCBI isolates.

**Fig. 2:**
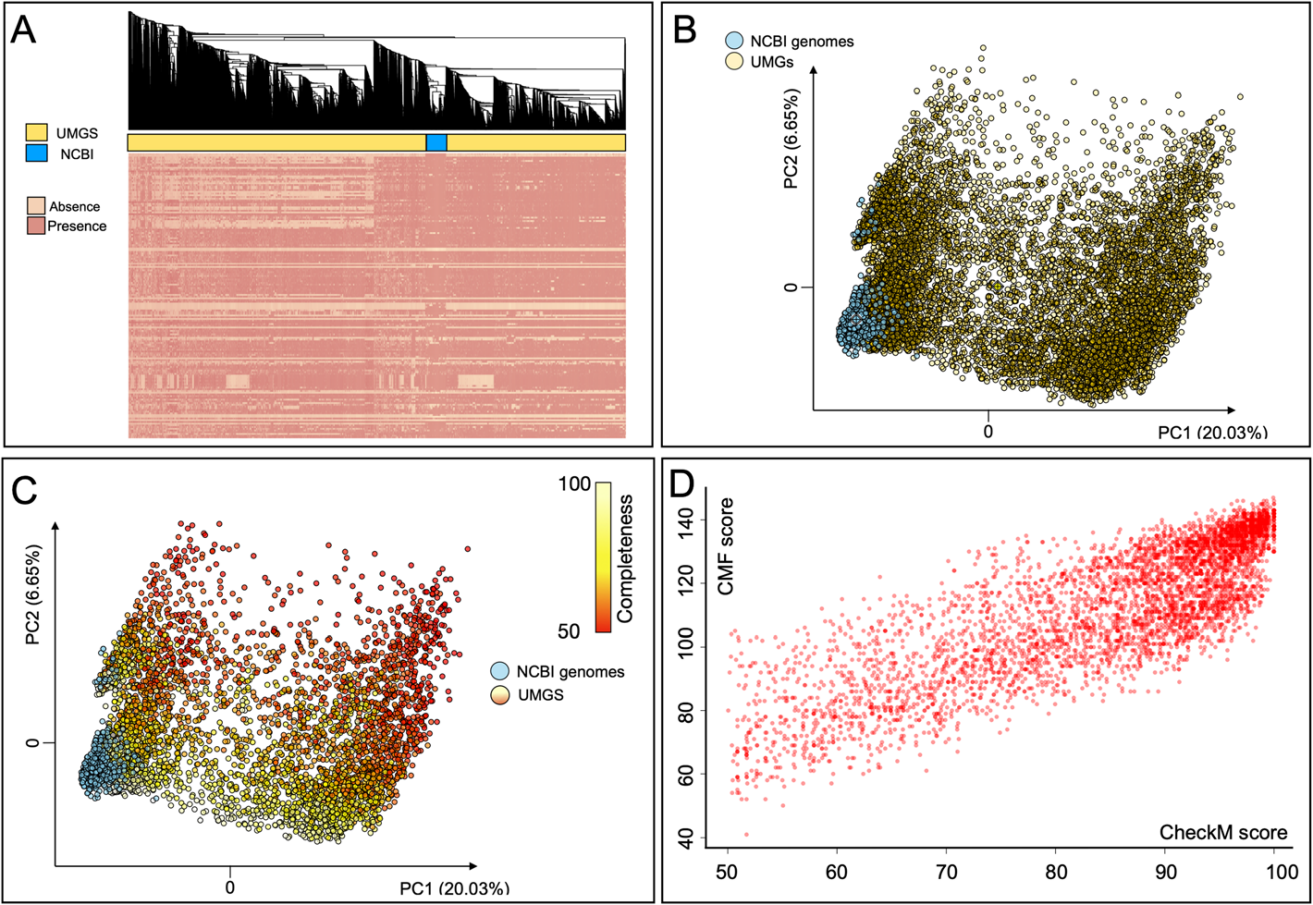
**a** Genomes clustering based on the presence/absence profile of CMF genes. The two generated clusters are highlighted by gold and skyblue underlying vectors for UMGS and NCBI genomes, respectively. The separation between the two groups is statistically significant (*P* < 0.001, Fisher’s exact test). **b** PCA based on Euclidean distances showing a significant separation between NCBI (skyblue) and UMGS (gold) genomes according to the presence/absence profile of CMF genes (*P* < 0.001, permutation test with pseudo-F ratio). **c** PCA based on Euclidean distances as presented in panel B) color-coded accordingly to the CheckM score of the analyzed genomes. A gradient of CheckM variation across the two-dimensional space is highlighted. **d** Biplot showing the significant correlation between CMF (Y- axis) and CheckM scores (X-axis) for the UMGS analyzed (red dots)

Clustering analysis and PCA of the presence/absence profiles of CMF genes in NCBI and UMGS genomes showed a segregation between the two groups of genomes (PCA based on Euclidean distances and Logistic PCA are provided in Fig. 2a-b and Additional File 5, respectively). Clustering analysis clearly separates the genome batch in two parts, demarking a difference between the two types of genomes (*P* < 0.001, Fisher’s exact test), with UMGS grouped on the left and right sides of the heatmap, flanking the NCBI genomes (Fig. 2a). In the same graphics, it is possible to notice how UMGS genomes systematically lack more genes when compared to NCBI genomes (32% ± 34.5 and 11.5% ± 24.2 genomes missing for a single CMF gene in UMGS and NCBI set, respectively). The PCA analysis carried out using the binary Euclidean metric showed a separation of the genomes in the two-dimensional plan (*P* < 0.001, permutation test with pseudo-F ratio), with NCBI genomes less disperse if compared to UMGS, indicating a more homogeneous representation of the CMF genes inside the NCBI group (Fig. 2b).

The genome quality of the 10.000 UMGS we obtained by retrieving the respective CheckM score. Result were superimposed on the same PCA (Fig. 2c), were UMGS are color-coded according to the corresponding CheckM value. Interestingly, our analysis highlighted a gradient of CheckM variation across the two-dimensional space, indicating a gradient of decreasing genomes completeness along the PC2 component. Confirming this observation, CheckM score is also correlated significantly (*P* < 0.0001) and negatively with PC2 component, showing how the completeness of the UMGS has gradually decreased as the axis index increases. Finally, a positive correlation between the CMF hits and the CheckM score in the 10.000 UMGS was observed (Kendall’s correlation tau = 0.6, *P* < 0.001) (Fig. 2d).

## Discussion

The reconstruction of bacterial genomes starting from short metagenomic sequences is certainly a complex process, both from a conceptual and a practical point of view. It is no wonder that among the missing genes the 16S rRNA, fundamental for bacterial life, has not been retrieved in 8034 out of 10,000 UMGS. This recurrent lack is probably inherent to the structure of the 16S rRNA gene, consisting in conserved and variable region and an overall similarity that can be up to 97% between two different bacterial species, making the assembly very hard in metagenomic data from microbial community [13]. On the other hand, fgocusing on other specific functionalities, it has been possible to highlight a systematic absence of genes encoding for ATP-dependent metalloprotease FtsH, DNA topoisomerase subunit 4, Dihydrolipolysine-residue acetyltransferase and Histidine biosynthesis protein HisB in the genomes of the analyzed UMAGs – missing in more than 85% of the UMGS genomes. This group of 4 genes represent a set of biologically diverse functions: ATP-dependent metalloprotease FtsH gene encodes for a metalloprotease that plays a crucial role in the control of membrane protein integrity and regulates LPS biosynthesis [14] and DNA topoisomerase subunit 4 gene encodes for a protein crucial in the chromosome segregation process, decatenating newly replicated chromosomes [15]. Particularly, the lack of this latter gene can lead to the impossibility for a bacterium to resolve DNA supercoilings, compromising the viability of the organism [16]. On the other hand, Dihydrolipolysine-residue acetyltransferase is a component of the pyruvate dehydrogenase complex and its absence in the UMGS genomes can suggest their strict anaerobic propensity. Finally, Histidine biosynthesis protein HisB is a protein involved in step 6 and 8 of the sub-pathway that synthesizes L-histidine from 5-phospho-alpha-D-ribose 1-diphosphate. The function of this protein is crucial for bacterial life, since histidine is required for multiple biological processes [17]. However, the lack of genes involved in histidine metabolism may indicate species variants auxotrophic for Histidine, possibly occurring in a highly syntrophic ecosystem as the human gut [17, 18].

## Conclusion

The present report provides a first attempt at screening both newly proposed UMGS and isolated genomes for the presence of a minimal set of core functionalities necessary but not sufficient for life. Our results showed that UMGS were substantially depleted in several essential genetic functions according to the CMF, including a recurrent depletion of 4 essential genes in more than the 85% of the UMGS, encoding for ATP-dependent metalloprotease FtsH, DNA topoisomerase subunit 4, Dihydrolipolysine-residue acetyltransferase and Histidine biosynthesis protein HisB. Even if a completeness of the 10.000 analyzed UMGS is greater than 50%, including 1.175 MAGs at median completeness of up to 96.5% and 893 MAGs complete at 77.8%, our data suggest that a relevant fraction of these genomes is missing genes encoding for essential functionalities to support life, thus raising possible concerns about their extrapolated biology. In certain circumstances, this feature is probably due to the inherent structure of certain genes, such as the 16S rRNA, which results in intrinsic difficulties in the assembly process. Differently, in other circumstances, the lack of some core genes may be due to the high degree of syntrophic shared by intestinal microbes, that may result in specialization and genome shrinkages. Furthermore, it is important to highlight that the CMF should not be considered the ‘only and true set of minimal functions’, since the two available minimal genomes don’t have a 100% overlap, and more minimal genomes are expected to be produced in the next time. In spite of that, some key functionalities which are systematically lacking in the UMGS – eg. DNA topoisomerase subunit 4 – pose some concerns about the possibility that UMGs may use alternative systems for compensating the lack of functions, which are currently not in our knowledge. Despite being an important evolutionary aspect [19], the systematic recurrence and number of missed functionalities in some of the UMGS suggest caution when interpreting their metabolic and ecological propensity on the basis on their peculiar profile of gene relative abundance, since we still miss key information about their basic functionalities for the support of autonomous life. Our analysis also points out the need to confirm the recovery of UMGS on metagenomic datasets obtained using third generation sequencing platforms providing longer reads, which have shown to aid genome completeness in de novo assembly and preserve more genomic information useful for species-level taxonomic assignment, such as operon structures [20]. Further studies also comprising different UMGs from different niches, the inclusion of a larger number of NCBI genomes and the stratification of the MAGs analyzed basing on their reported completeness, are needed to verify and refine the accuracy of our results.

## Methods

The minimal genomes JCVI-syn 3.0 [11] and *C. Eth-2.0* [12] were downloaded from NCBI website and annotated using prokka 1.13.3 standard pipeline and –addgenes flag [21] in a Unix CentOS environment. Genes assigned with certainty (not flagged with ‘hypothetical’ or ‘putative’ and manually screened) were retained for the creation of the CMF. A total of 400 NCBI and 10,000 metagenome-assembled genomes have been downloaded from the “Assembly” page of NCBI (the parameters “Complete” and “Representative” were selected) and from the European Nucleotide Archive under study ID PRJEB26432, respectively, and annotated as reported above for the synthetic genomes. The NCBI genomes selected were manually selected and bacteria isolate from the gut environment were selected, while the MAGs genomes were randomly chosen since the latter are all from the same ecosystem (human gut). For each, the presence/absence profile of CMF was obtained comparing the detected functions among each genome and the CMF, retaining only the matching hits. Clustering of the CMF presence/absence profiles of NCBI and UMGS genomes was performed in R studio (version 1.2.1355 - R version 3.5.1 [22]), using the Jaccard binary distance and the Ward’s minimal variance clustering method (packages ‘stats’ V3.6.0 [15] and ‘gplot’ V3.0.1.1 [23]). Finally, the Euclidean distances between the CMF presence/absence profiles of UMGS and NCBI genomes were calculated and a multivariate analysis was carried out using the vegan package (V2.5–5 [24]). The separation between the NCBI genomes and the UMGS in the two-dimensional space was verified using a permutation test with pseudo-F ratio (ADONIS function of the ‘vegan’ package).

## Supplementary information

**Additional file 1.** List of genes included in CMF. For each gene the KEGG orthology is detailed, with three functional levels reported whenever possible. CMF genes are grouped based on the lower level of KEGG orthology available.

**Additional file 2.** Detailed statistics of the analyzed genomes and the CMF functions. For the 400 NCBI isolates genomes species name is reported. For the 10,000 genomes retrieved from Almeida et al. study the values of completeness and contamination and relative area plots are reported.

**Additional file 3.** Pie charts representing the phyla distribution in the two cohorts of genomes analyzed (A for NCBI genomes, B for UMGS).

**Additional file 4.** Boxplots representing the percentage of CMF genes covered by different groups of genomes: UMGS having a CheckM completeness score higher or equal than 90% (dark red), UMGS having a CheckM completeness score lower than 90% (red) and NCBI isolates genomes (skyblue).

**Additional file 5. **Logistic PCA of the of the presence/absence profiles of CMF genes in NCBI and UMGS genomes. A significant separation between NCBI (skyblue) and UMGS (gold to red gradient based on CheckM completeness score) genomes was obtained (*P* < 0.001, permutation test with pseudo-F ratio).

## Data Availability

The datasets analyzed during the current study are available in the ENA repository, https://www.ebi.ac.uk/ena/data/view/PRJEB26432&portal=wgs_set, and in the NCBI repository, https://www.ncbi.nlm.nih.gov/assembly/?term. JCVI-Syn3.0 and *C. Eth-2.0* genomes were downloaded from NCBI Genbank using their relative accession numbers CP014940 (https://www.ncbi.nlm.nih.gov/nuccore/CP014940) and CP035535 (https://www.ncbi.nlm.nih.gov/nuccore/CP035535).

## Abbreviations

CMF: Core gene set of Minimal Functions
GM: Gut Microbiota
HGR: Human Gut Reference
HR: Human-specific Reference
MAGs: Metagenome-Assembled Genomes
PCA: Principle Components Analysis
UMGS: Unknown MetaGenomic Species
