# Supplementary material for: Do the human gut metagenomic species possess the minimal set of core functionalities necessary for life?
Source: BMC Genomics. 2020 Sep 30;21:678. doi: 10.1186/s12864-020-07087-8 (PMC7525937; doi:10.1186/s12864-020-07087-8)
Supplement: Supplementary file 1 — Additional file 1. List of genes included in CMF. For each gene the KEGG orthology is detailed, with three functional levels reported whenever possible. CMF genes are grouped based on the lower level of KEGG orthology available. [file 12864_2020_7087_MOESM1_ESM.pdf]

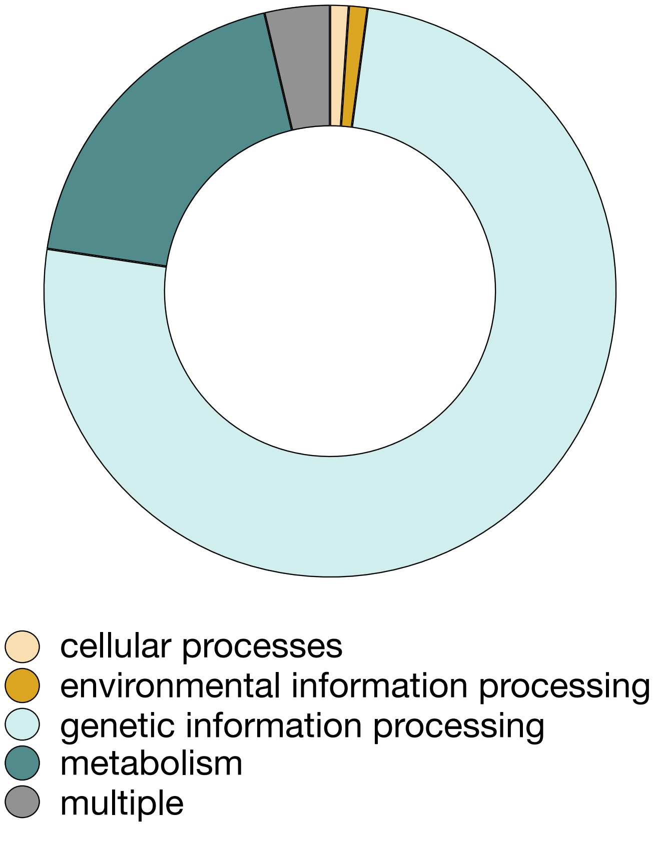
cellular processes
environmental information processing
genetic information processing
metabolism
multiple
