# Supplementary figures and images for: Do the human gut metagenomic species possess the minimal set of core functionalities necessary for life?
Source: BMC Genomics. 2020 Sep 30;21:678. doi: 10.1186/s12864-020-07087-8 (PMC7525937; doi:10.1186/s12864-020-07087-8)

A

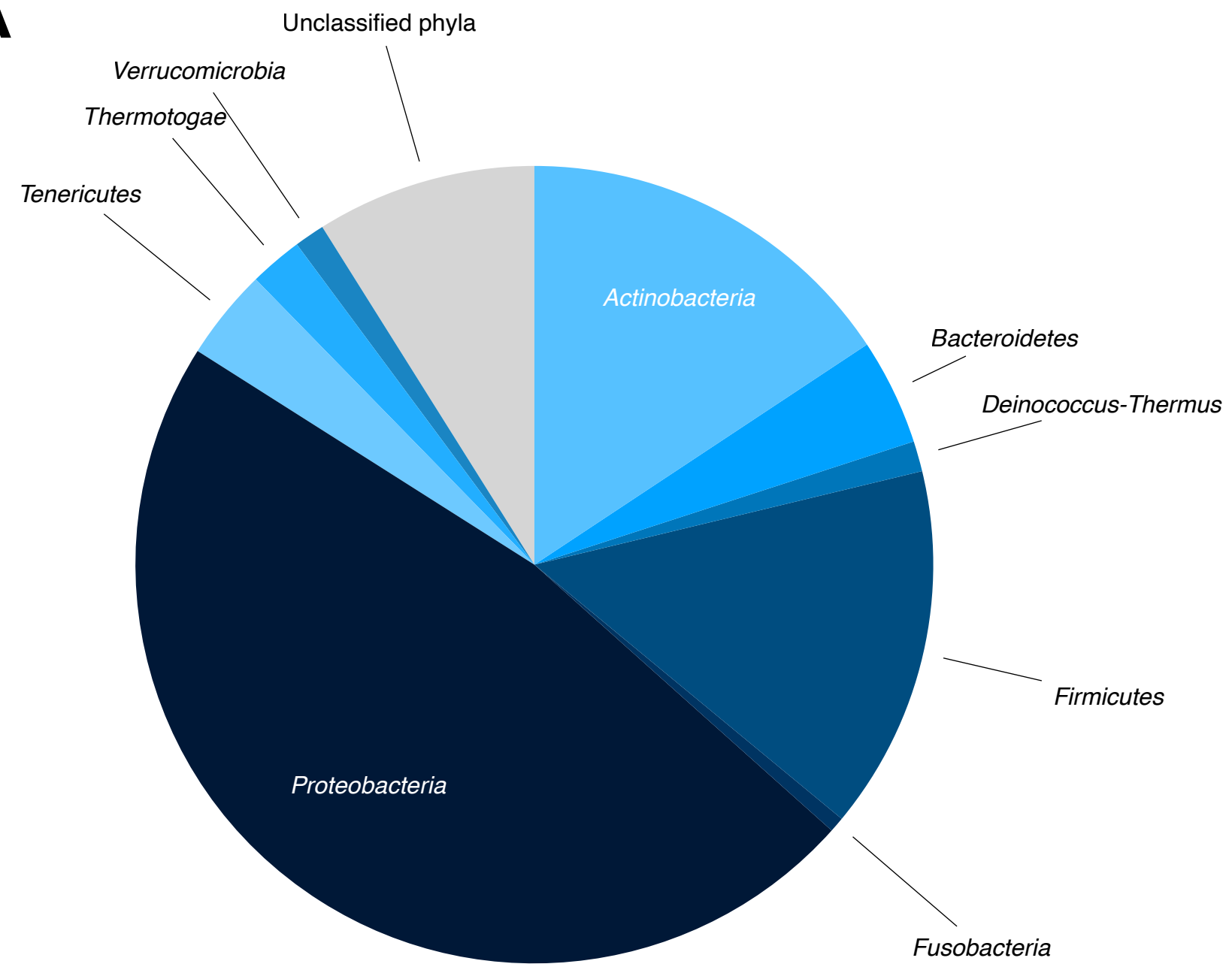

B

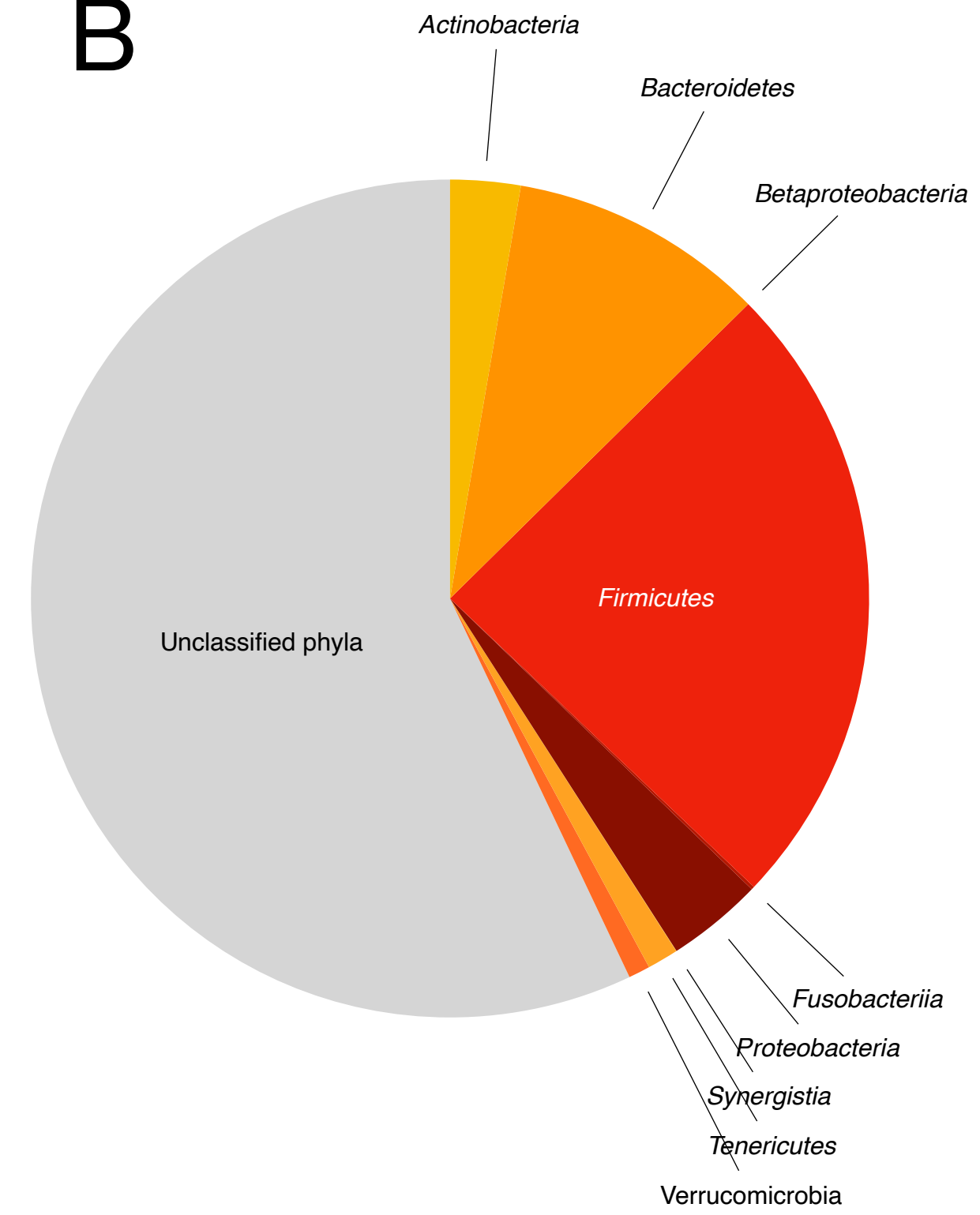

Supplement: Supplementary file 3 — Additional file 3. Pie charts representing the phyla distribution in the two cohorts of genomes analyzed (A for NCBI genomes, B for UMGS). [file 12864_2020_7087_MOESM3_ESM.pdf]

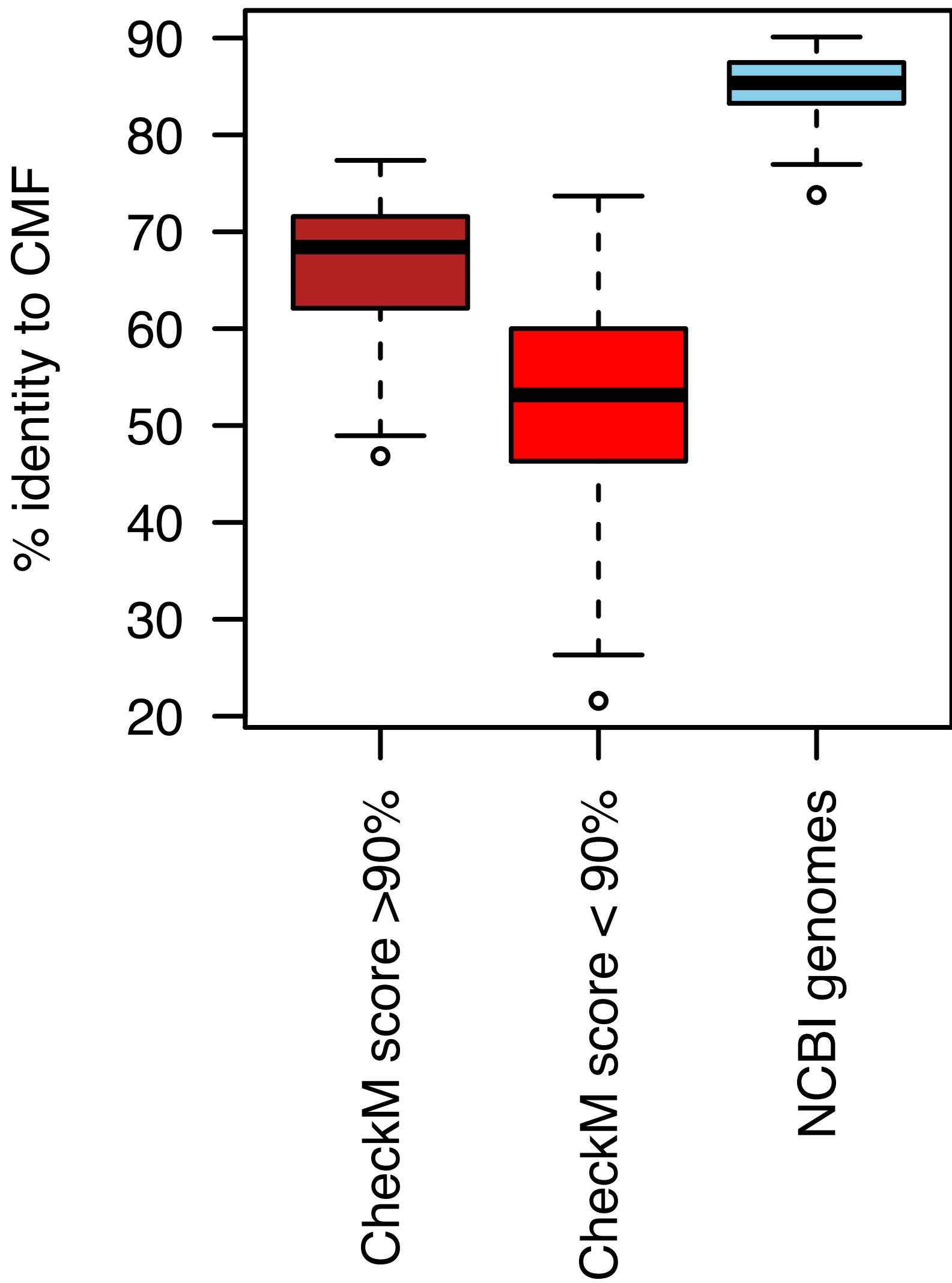

Supplement: Supplementary file 4 — Additional file 4. Boxplots representing the percentage of CMF genes covered by different groups of genomes: UMGS having a CheckM completeness score higher or equal than 90% (dark red), UMGS having a CheckM completeness score lower than 90% (red) and NCBI isolates genomes (skyblue). [file 12864_2020_7087_MOESM4_ESM.pdf]

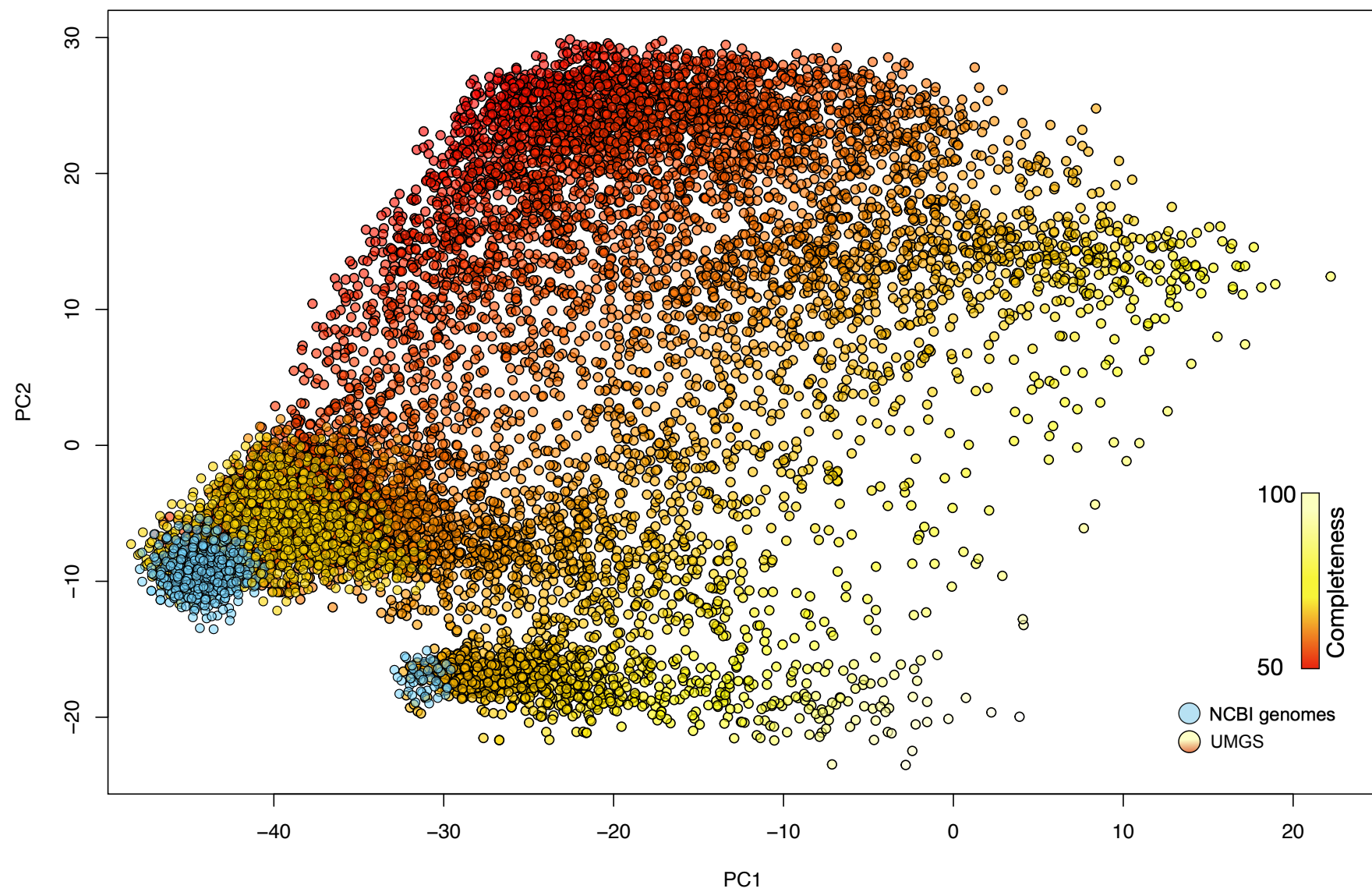

Supplement: Supplementary file 5 — Additional file 5. Logistic PCA of the of the presence/absence profiles of CMF genes in NCBI and UMGS genomes. A significant separation between NCBI (skyblue) and UMGS (gold to red gradient based on CheckM completeness score) genomes was obtained (P < 0.001, permutation test with pseudo-F ratio). [file 12864_2020_7087_MOESM5_ESM.pdf]
